# Vaccination with Recombinant Aspartic Hemoglobinase Reduces Parasite Load and Blood Loss after Hookworm Infection in Dogs

**DOI:** 10.1371/journal.pmed.0020295

**Published:** 2005-10-04

**Authors:** Alex Loukas, Jeffrey M Bethony, Susana Mendez, Ricardo T Fujiwara, Gaddam Narsa Goud, Najju Ranjit, Bin Zhan, Karen Jones, Maria Elena Bottazzi, Peter J Hotez

**Affiliations:** 1Division of Infectious Diseases and Immunology, Queensland Institute of Medical Research, Brisbane, Queensland, Australia,; **2**Department of Microbiology and Tropical Medicine, The George Washington University Medical Center, Washington, District of Columbia, United States of America; Leiden University Medical Centerthe Netherlands

## Abstract

**Background:**

Hookworms infect 730 million people in developing countries where they are a leading cause of intestinal blood loss and iron-deficiency anemia. At the site of attachment to the host, adult hookworms ingest blood and lyse the erythrocytes to release hemoglobin. The parasites subsequently digest hemoglobin in their intestines using a cascade of proteolysis that begins with the *Ancylostoma caninum* aspartic protease 1, APR-1.

**Methods and Findings:**

We show that vaccination of dogs with recombinant *Ac-*APR-1 induced antibody and cellular responses and resulted in significantly reduced hookworm burdens (*p* = 0.056) and fecal egg counts (*p* = 0.018) in vaccinated dogs compared to control dogs after challenge with infective larvae of *A. caninum*. Most importantly, vaccinated dogs were protected against blood loss (*p* = 0.049) and most did not develop anemia, the major pathologic sequela of hookworm disease. IgG from vaccinated animals decreased the catalytic activity of the recombinant enzyme in vitro and the antibody bound in situ to the intestines of worms recovered from vaccinated dogs, implying that the vaccine interferes with the parasite's ability to digest blood.

**Conclusion:**

To the best of our knowledge, this is the first report of a recombinant vaccine from a hematophagous parasite that significantly reduces both parasite load and blood loss, and it supports the development of APR-1 as a human hookworm vaccine.

## Abstract 2

Vaccination of dogs with a recombinant protease produced by hookworms can reduce blood loss when these dogs are infected with the hookworm *Ancylostoma caninum.*

## Introduction

Hookworms infect more than 700 million people in tropical and subtropical regions of the world. The major species infecting humans are *Necator americanus* and *Ancylostoma duodenale.* The parasites feed on blood, causing iron-deficiency anemia, and as such, are a major cause of disease burden in developing countries [1]. Unlike other human helminthiases, worm burdens do not generally decrease with age; in fact, recent findings revealed that the heaviest worm burdens are found among the elderly [2,3]. Whereas anthelminthic chemotherapy with benzimidazole drugs is effective in eliminating existing adult parasites, re-infection occurs rapidly after treatment [4], making a vaccine against hookworm disease a desirable goal.

Canines can be successfully vaccinated against infection with the dog hookworm, *Ancylostoma caninum,* by immunization with third-stage infective larvae (L3) that have been attenuated with ionizing radiation [5–7]. Subsequently, varying levels of vaccine efficacy have been reported for the major antigens secreted by hookworm L3 using hamsters [8,9] and dogs [10]. Despite obtaining encouraging levels of protection with larval antigens, only partial reductions in parasite load (fecal egg counts and adult worm burdens) were reported. Moreover, protective antigens from the larval stage are only expressed by L3, and not adult worms, rendering antibodies against these L3 secretions useless against parasites that have successfully reached adulthood in the gut and begun to feed on blood. We therefore suggest that an ideal hookworm vaccine would require a cocktail of two recombinant proteins, one targeting the infective larva and the second targeting the blood-feeding adult stage of the parasite [11].

Of the different families of proteins expressed by blood-feeding parasitic helminths, proteolytic enzymes have shown promise as intervention targets for vaccine development [12,13]. Proteases are pivotal for a parasitic existence, mediating fundamental physiologic processes such as molting, tissue invasion, feeding, embryogenesis, and evasion of host immune responses [12,14]. Parasite extracts enriched for proteases protect sheep against the blood-feeding nematodes *Haemonchus contortus* [15–18] and *Ostertagia ostertagi* [19]; however, significant protective efficacy has not been shown with a purified recombinant protease from nematodes of livestock.

Hookworms feed by burying their anterior ends in the intestinal mucosa of the host, rupturing capillaries and ingesting the liberated blood. Erythrocytes are lysed by pore formation [20], releasing hemoglobin (Hb) into the lumen of the parasite's intestine, where it is degraded by a semi-ordered pathway of catalysis that involves aspartic, cysteine, and metalloproteases [21]. Vaccination of dogs with a catalytically active recombinant cysteine hemoglobinase, *Ac*-CP-2, induced antibodies that neutralized proteolytic activity and provided partial protection to vaccinees by reducing egg output (a measure of intestinal worm burden) and worm size, but significant reductions of adult worm burdens and/or blood loss were not observed [22]. Anemia is the primary pathology associated with hookworm infection, and an ultimate human hookworm vaccine would limit the amount of blood loss caused by feeding worms and maintain normal levels of Hb. This is particularly important in young children as well as women of child-bearing age, in whom menstrual, and particularly fetal, Hb demands are considerable, rendering these populations most vulnerable to the parasite [1].

Here we describe vaccination of dogs with the aspartic hemoglobinase of *A. caninum, Ac-*APR-1 [21,23] and show that vaccination resulted in the production of neutralizing antibodies, significantly reduced egg counts, and significantly reduced adult worm burdens. Most importantly, Hb levels of vaccinated dogs were significantly higher than those of dogs that were vaccinated with adjuvant alone after parasite challenge. These data show that aspartic hemoglobinases, particularly APR-1, are efficacious vaccines against canine hookworm disease, providing strong support for further investigation and development of APR-1 as a recombinant vaccine against human hookworm disease.

## Methods

### Expression of Recombinant *Ac-*APR-1 in *Pichia pastoris*

The entire open reading frame of *Ac-*APR-1 encoding the zymogen (spanning Ser-17 to the C-terminal Phe-446) but excluding the predicted signal peptide was cloned into the expression vector pPIC-Zα (Invitrogen, Carlsbad, California, United States) using the XbaI and EcoRI sites. Yeast, *P. pastoris* X 33, was transformed with the vector encoding the *Ac-*APR-1 zymogen as recommended by the manufacturer (Invitrogen) with modifications. Protein disulfide isomerase (PDI) gene in the vector pPIC3.5 (a gift from Mehmet Inan, University of Nebraska, Lincoln, Nebraska, United States) was cut with SacI and transformed into *P. pastoris* X 33 cells which were already transformed with *Ac-apr-1* following the manufacturer's instructions. Eight transformed colonies were picked from YPD plates containing Geneticin (0.5–1.0 mg·ml^−1^) and Zeocin (1.0 mg·ml^−1^) and tested for *Ac*-APR-1 expression following the manufacturer's instructions. The highest expressing colony was selected and transferred to suspension culture in flasks containing BMG medium (buffered minimal glycerol: 1.34% yeast nitrogen base, 0.00004% d-biotin, 1% w/v glycerol, and 100 mM potassium phosphate, [pH 6.0]). Suspension cultures were then transferred to a Bioflo 3000 fermentor (New Brunswick Scientific, Edison, New Jersey, United States) utilizing a 5-l vessel as described [8]. The recombinant protein was secreted into culture medium and affinity purified on nickel-agarose as described elsewhere [8]. Progress of purification was monitored using SDS-PAGE gels stained with Coomassie Brilliant Blue and immunoblots using monoclonal antibodies to the vector-derived *myc* epitope. Recombinant *Ac-*APR-1 was treated with PNGase F and O-glycosidase, according to the manufacturer's instructions (Enzymatic CarboRelease kit; QA-Bio, San Mateo, California, United States), under denaturing conditions to remove any N-linked and O-linked oligosaccharides. Deglycosylation was performed only to confirm the presence of N-linked sugars on the recombinant molecule. All remaining studies were conducted with the glycoprotein.

### Activation and Hemoglobinolytic Activity of Recombinant APR-1

The unactivated zymogen was used for vaccination. A small amount of the purified protein, however, was buffer exchanged into 100 mM sodium formate (pH 3.6)/0.15 M NaCl using a PD10 desalting column (Amersham Biosciences, Little Chalfont, United Kingdom) to facilitate proteolytic activation and removal of the pro-region. One microgram of purified, activated protease was then added to 10 μg of dog Hb in the same buffer and incubated at 37 °C for 2 h. Cleavage of Hb was assessed visually by staining SDS-PAGE gels with Coomassie Brilliant Blue.

### Animal Husbandry

Purpose-bred, parasite naive, male beagles aged 8 ± 1 wk were purchased from Marshall Farms (North Rose, New York, United States), identified by ear tattoo, and maintained in the George Washington University Animal Research Facility as previously described [24]. The experiments were conducted according to a protocol approved by the University Animal Care and Use Committee (IACUC 48–12,0 [12,1]E). Before the first vaccination and after each subsequent one, a blood sample was obtained from each dog.

### Vaccine Study Design and Antigen-Adjuvant Formulation

The vaccine trial was designed to test *Ac-*APR-1 zymogen formulated with the adjuvant AS03 [25], obtained from GlaxoSmithKline (a kind gift from Drs. Joe Cohen and Sylvie Cayphas; GSK Biologicals, Rixensart, Belgium). To make six doses of *Ac*-APR-1 formulated with AS03, 600 μg of recombinant protein (1.5 ml of *Ac*-APR-1 at a concentration of 0.4 mg·ml^−1^) was mixed with 1.2 ml of 20 mM Tris-HCl, 0.5 M NaCl (pH 7.9), and 1.5 ml of AS03; the contents of the tube were vortex mixed for 30 sec then shaken at low speed for 10 min. Dogs were immunized with 100 μg of formulated antigen in a final volume of 0.5 ml. AS03-only control was prepared as described above, with PBS included instead of *Ac-*APR-1.

### Canine Immunizations and Antibody Measurements

Five beagles were immunized three times with AS03-formulated *Ac-*APR-1 by intramuscular injection. The vaccine was administered on days 0, 21, and 42, beginning when the dogs were 62 ± 4 d of age. As negative controls, five beagles were also injected intramuscularly with an equivalent amount of AS03 using the identical schedule. Blood was drawn at least once every 21 d and serum was separated from cells by centrifugation. Enzyme-linked immunosorbent assays (ELISA) were performed as previously described [24]. Recombinant *Ac-*APR-1 was coated onto microtiter plates at a concentration of 5.0 μg·ml^−1^. Dog sera were titrated between 1:100 and 1:2 × 10^6^ to determine endpoint titers (the highest dilution of test group [APR-1] sera that gave a mean O.D. of ≥3× the mean optical density (OD) of sera from the control group). Anti-canine IgG1, IgG2, and IgE antibodies conjugated to horseradish peroxidase (Bethyl Laboratories, Montgomery, Texas, United States) were used at a dilution of 1:1,000. Blood was collected from dogs before immunizations and 7 d after the third vaccination but before L3 challenge.

### Stimulation of and Cytokine Measurements from Cultured Whole Blood

Lymphoproliferation assays were performed using a whole blood microassay as previously described [26]. Briefly, 25 μl of heparinized blood was diluted in 200 μl of RPMI 1640 medium (Gibco, Invitrogen) supplemented with 3% antibiotic/antimycotic solution (Gibco). All tests were performed in triplicate in 96-well flat-bottomed culture plates using recombinant APR-1 at a concentration of 25 μg·ml^−1^ and concanavalin A (ConA; Sigma-Aldrich, St. Louis, Missouri, United States) at 80 μg·ml^−1^. Incubation was carried out in a humidified 5% CO_2_ atmosphere at 37 °C for 2 d (ConA-stimulated cultures) and 5 d (APR-1). Cells were pulsed for 6 h with 1.0 μCi of [^3^H] thymidine (PerkinElmer Life And Analytical Sciences, Boston, Massachusetts, United States) and harvested onto glass fiber filters. Radioactive incorporation was determined by liquid scintillation spectrometry. Proliferation responses were expressed as stimulation indices, SI (where SI = mean proliferation of stimulated cultures/mean proliferation of unstimulated cultures). For cytokine analyses, whole blood (collected as described above) was diluted 1:8 in RPMI supplemented with 3% antibiotic/antimycotic solution in a 48-well flat-bottomed culture plate with a final volume of 1.0 ml per well. Cells were stimulated by the addition of 25 μg·ml^−1^ of recombinant APR-1. After 48 h of incubation at 37 °C, 700 μl of supernatant was removed from each well and stored at −20 °C until required for the cytokine assay. IL-4, IL-10, and IFN-γ were measured using a capture ELISA assay for dogs (R & D Systems, Minneapolis, Minnesota, United States) following the manufacturer's instructions. Biotin-labeled detection antibodies were used (100 ng·ml^−1^), revealed with streptavidin-HRP (Amersham Biosciences), and plates were developed with OPD (O-Phenylenediamine) substrate system (Sigma-Aldrich).

### Hb Measurements

To determine Hb concentrations of experimental dogs, 1–2 ml of blood were collected in EDTA and analyzed using a QBC VetAutoread Hematology System and VETTEST Software (IDEXX Laboratories, Westbrook, Maine, United States).

### Hookworm Infections and Parasite Recovery

Two weeks after the final immunization, dogs were anaesthetized using a combination of ketamine and xylazine (20 mg·kg^−1^ and 10 mg·kg^−1^ respectively) and infected via the footpad with 500 *A. caninum* L3 as described elsewhere [22]. Quantitative hookworm egg counts (McMaster technique) were obtained for each dog 3 d per wk from days 12–26 postinfection. Four weeks postinfection, the dogs were killed by intravenous injection of barbiturate, and adult hookworms were recovered and counted from the small and large intestines at necropsy [24]. The sex of each adult worm was determined as described elsewhere [8]. Approximately 1–2 cm lengths of small intestine were removed and stored in formalin for future histopathologic analysis.

### Statistical Methods

In most cases, the small size of the samples did not enable us to determine if values were normally distributed, so the following non-parametric tests were used: Mann-Whitney *U* was used to test whether two independent samples (groups) came from the same population, and the Kruskal Wallis *H* test was used to determine if several independent samples came from the same population. Normally distributed variables were tested in the following manner: The independent-samples *t*-test procedure was used to compare the means for two groups, and an analysis of variance was used to test the hypothesis that several means are equal, followed by a Dunnet post hoc multiple comparison *t*-test to compare the vaccine treatment groups against the control group. Differences were considered statistically significant if the calculated *p-*value was equal to or less than 0.1 (two-sided). The percentage reduction or increase in adult hookworm burden in the vaccinated group was expressed relative to the control group as described elsewhere [24].

### Immunohistochemistry

Adult hookworms were recovered at necropsy from vaccinated dogs and control dogs, washed briefly, then fixed and sectioned as previously described [22]. To observe whether IgG from vaccinated but not control dogs, bound to APR-1 lining the intestinal microvillar surface of worms in situ, sections were probed with Cy3-conjugated rabbit anti-dog IgG (Jackson Immunoresearch, West Grove, Pennsylvania, United States) at a dilution of 1:500 as described elsewhere [27]. Sections were visualized using a Leica IM 100 inverted fluorescence microscope (Leica Microsystems, Wetzlar, Germany).

### Effect of Anti–*Ac*-APR-1 IgG on Proteolytic Activity

Canine IgG was purified from sera of vaccinated dogs using protein A-agarose (Amersham Biosciences) as previously described [23]. Purified IgG (0.2 μg) was incubated with 1.0 μg of recombinant *Ac-*APR-1 for 45 mins prior to assessing catalytic activity of APR-1 against the fluorogenic substrate o-aminobenzoyl-IEF-nFRL-NH_2_ as described previously [23]. The aspartic protease inhibitor, pepstatin A, was included at a final concentration of 1.0 μM as a positive control for enzymatic inhibition. Data was recorded from triplicate experiments and presented as relative fluorescence units using a TD700 fluorometer (Turner Designs, Sunnyvale, California, United States).

## Results

### Secretion of Catalytically Active *Ac-*APR-1 by *P. pastoris*

Yeast secreted the APR-1 zymogen into culture medium at an approximate concentration of 1.0 mg·l^−1^ (Figure 1A). In the absence of co-expression with the PDI chaperone, the amount of APR-1 secreted by *P. pastoris* was approximately half that obtained here (not shown). *Ac-*APR-1 has one potential glycosylation site at Asn-29 of the zymogen (after removal of the signal peptide), and treatment with PNGase F decreased the size of the recombinant protein by the expected size (2–3 kDa; not shown). The activated recombinant protease readily digested canine Hb at acidic pH (Figure 1B), confirming that *Ac-*APR-1 expressed in yeast is catalytically active and digested Hb with similar efficiency to recombinant *Ac-*APR-1 produced in baculovirus (data not shown).

**Figure 1:**
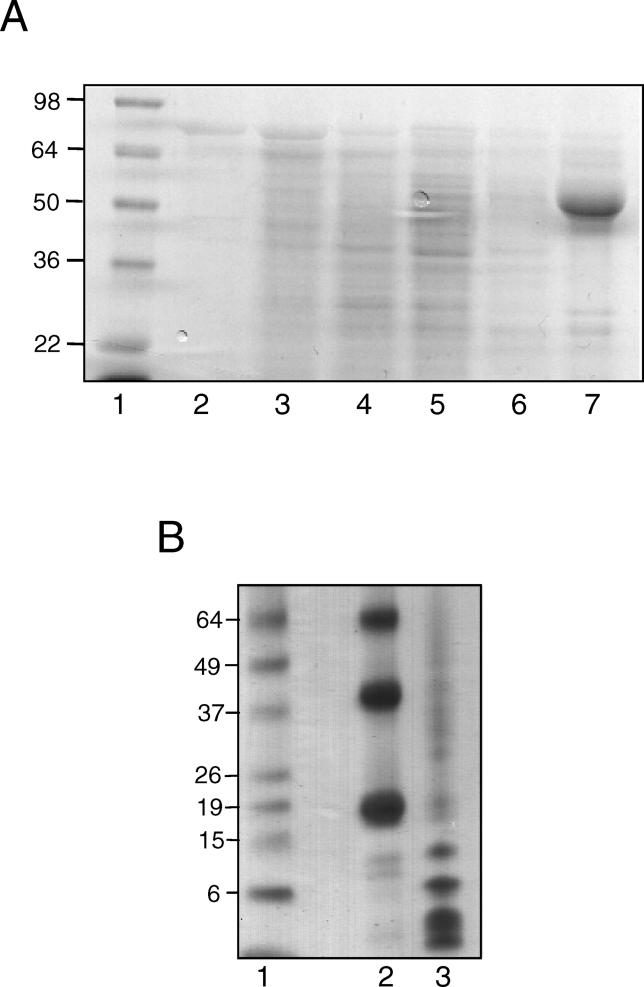
P. pastoris Secrete *Ac-*APR-1 Zymogen that Autoactivates at Low pH and Degrades Canine Hb SDS-PAGE gel stained with Coomassie Brilliant Blue showing purification of recombinant APR-1 zymogen from *P. pastoris* culture supernatant. (A) Lane 1, molecular weight markers; lane 2, concentrated culture supernatant; lane 3, flow-through from a nickel-IDA column; lane 4, 5 mM imidazole wash; lane 5, 20 mM imidazole column eluate; lane 6, 60 mM imidazole eluate; and lane 7, 1 M imidazole eluate. Purified recombinant APR-1 zymogen was activated by buffer exchange into 0.1 M sodium formate/0.1 M NaCl (pH 3.6). (B) Lane 1, molecular weight markers; lane 2, 5.0 μg of canine Hb (pH 3.6); and lane 3, 5.0 μg of canine Hb (pH 3.6) incubated with 0.2 μg of recombinant APR-1.

### Recombinant *Ac-*APR-1 Is Immunogenic in Dogs

AS03 was used as an adjuvant based on its ability to induce a higher IgG1 response and greater reduction in hookworm egg counts when used to vaccinate dogs in a head-to-head comparison of a cysteine hemoglobinase formulated with four different adjuvants [22]. Dogs immunized with recombinant *Ac*-APR-1 formulated with AS03 produced IgG1 and IgG2 antibody responses as measured by ELISA using the recombinant protein (Figure 2). IgE titers were low (<1:1,500) and were not sustained past challenge. We did not adsorb IgG from serum before measuring IgE in this study; however, in previous trials IgG was removed and we did not see a difference in antigen-specific IgE titers. For vaccinated dogs, maximum IgG2 titers of 1:121,500 were attained by all five dogs after the second vaccination. High titers persisted through challenge and decreased to 1:26,098 by necropsy. IgG1 titers peaked at 1:13,500 after the third vaccination in all four dogs and dropped to 1:3,600 by necropsy. Dogs immunized with adjuvant alone did not generate detectable immune responses greater than 1:500, even after larval challenge.

**Figure 2:**
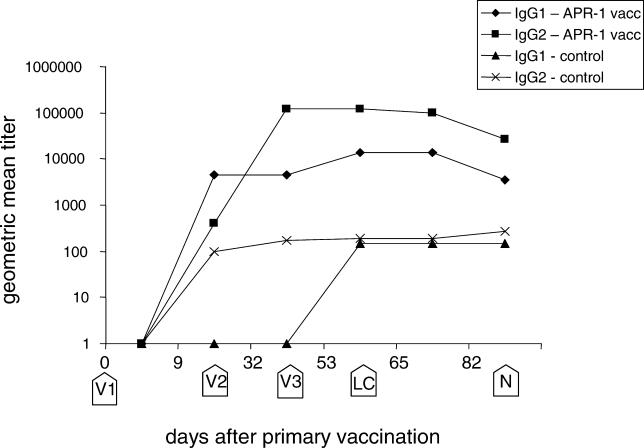
The Geometric Mean Titers of the IgG1 and IgG2 Antibody Responses of Dogs Vaccinated with Recombinant *Ac*-APR-1 Formulated with AS03 or AS03 Alone LC, day on which dogs were challenged with hookworm L3; N, day of necropsy; V1, V2, and V3, days on which animals were vaccinated.

Dogs rapidly acquire resistance to hookworm with maturity. A single dog was therefore removed from the control group (for all analyses) because its weight was greater than the acceptable range at all time points after the first vaccination (mean plus or minus three standard errors).

### Vaccination Induces Antigen-Specific Cell Proliferation and Cytokine Production

Vaccination with APR-1 induced a high level of lymphocyte/leukocyte proliferation compared with control dogs when cells were stimulated with APR-1 (*p* < 0.01, *t*-test). Cells from both vaccinated and control dogs proliferated equally when stimulated with mitogen (Figure 3A and 3B). No significant proliferation to APR-1 was observed before the immunization process. Immunization with APR-1 elicited antigen-specific production of IFN-γ (*p* = 0.03, *t-*test) (Figure 3C). In contrast, we did not detect significant production of IL-4 or IL-10 after stimulation with APR-1 in either vaccinated or control groups (not shown).

**Figure 3:**
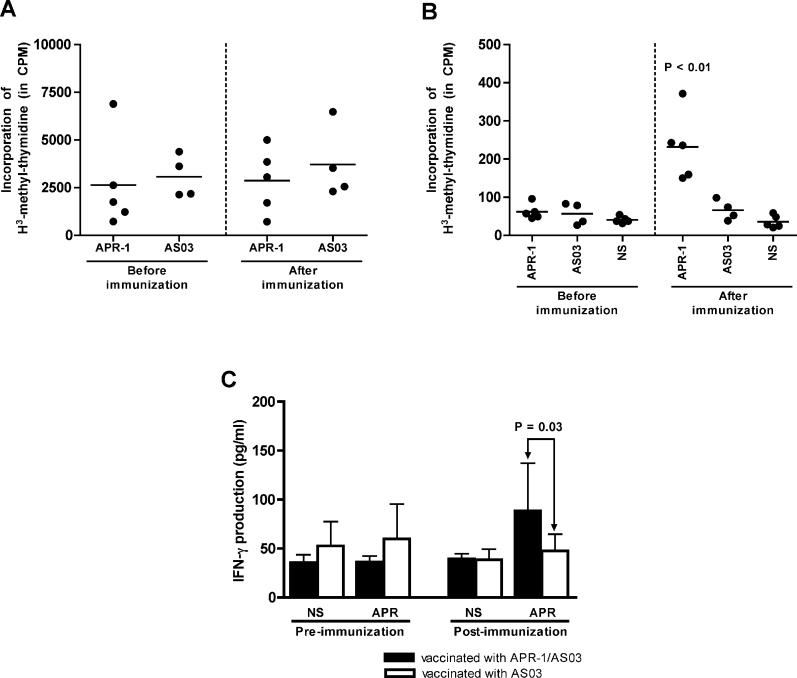
Canine Cellular Immune Response to Vaccination with Recombinant *Ac-*APR-1 Cell proliferation of whole blood cells from vaccinated (APR-1) and control dogs (AS03) when stimulated with concanavalin A (A) or recombinant *Ac-*APR-1 (B) before (day 0) and after the final immunization (day 51). The *p*-value comparing the mean differences between the vaccinated group and controls is denoted. Detection of secreted IFN-γ in whole blood cultures taken from vaccinated and control dogs before and after immunization (C). Mean cytokine concentrations are indicated in pg·ml^−1^ with standard error bars. Statistically significant differences are indicated above the bars by *p*-values. APR, stimulated with recombinant APR-1; NS, non-stimulated cultures.

### Vaccination with *Ac*-APR-1 Decreases Fecundity of Female Hookworms

Dogs develop age- and exposure-related immunity to *A. caninum* [5], so we therefore observed egg counts from vaccinated animals up to 26 d postchallenge, after which we often observe a significant decrease in egg counts in some dogs. Because of daily variation in egg counts from infected dogs (A. Loukas. S. Mendez, and P. Hotez, unpublished data), we analyzed the data in two ways. Firstly, the median egg counts for days 21, 23, and 26 postinfection were used to compare worm fecundity between vaccinated and control groups. A 70% decrease in median egg counts was observed in dogs vaccinated with *Ac-*APR-1 (2,650 eggs per gram of feces g]) compared with dogs that were vaccinated with adjuvant alone (8,725 epg) when median egg counts were calculated for the three time points measured after larval challenge (Figure 4A). We then compared geometric mean values of egg counts between the two groups (Figure 4B), and showed that mean egg counts of the vaccinated animals remained lower than the control animals as worms became fecund by day 21, implying that fecundity of female worms diminished significantly as they began to feed on blood containing anti–APR-1 antibodies. By day 26 postchallenge, there was an 85% reduction in mean egg counts between the two groups. For statistical analyses, we transformed egg counts into log values and ran the test in two ways: (1) comparing the log transformed epgs in the last three egg counts by analysis of variance (Kruskall-Wallis) revealed no significant differences among the groups for the last three egg counts when each time point was considered individually; and (2) comparing pooled data from the last three egg counts using a Mann-Whitney test (APR-1 versus control), revealed a statistically significant difference (*p* = 0.018).

**Figure 4:**
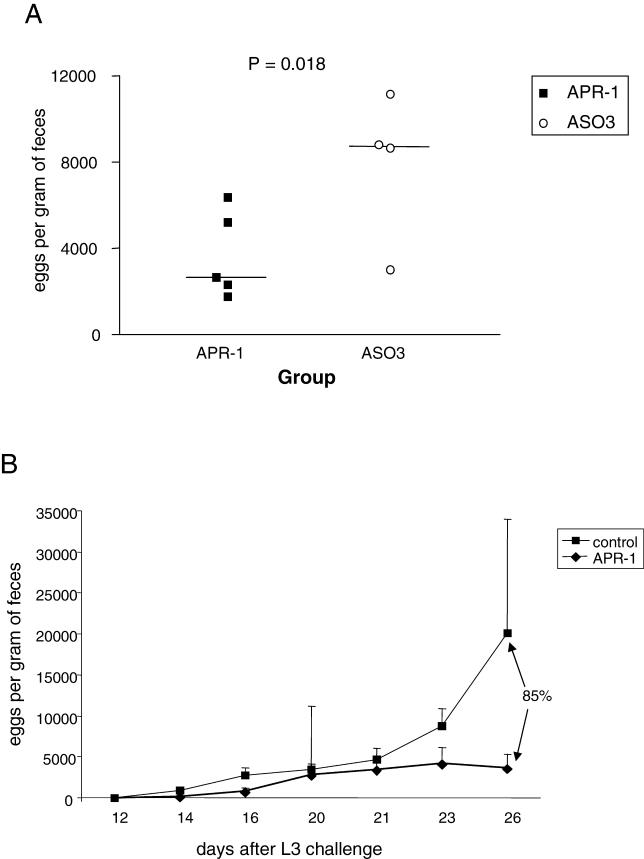
Vaccination with APR-1 Reduces Fecal Egg Counts of Dogs after Challenge Infection with Hookworms Statistically significant reduction (*p* = 0.018) in median fecal egg counts sampled on days 21, 23, and 26 of dogs vaccinated with APR-1 compared to dogs that received adjuvant alone. (A). Geometric mean values of fecal egg counts from vaccinated and control dogs between challenge infection and necropsy. (B). Error bars refer to the standard error of the mean.

### Vaccination with *Ac*-APR-1 Significantly Reduces Adult Hookworm Burdens

A statistically significant difference at the *p* ≤ 0.1 level (*p* = 0.095; Mann-Whitney *U* test) was detected for a one-sided test between median adult worm burdens recovered from vaccinated dogs (182) compared with control dogs (270) but not for a two-sided test (*p* = 0.190) (Figure 5). Percentage reduction of the median worm counts was 33% when data from both sexes of worms were combined, 30% for male worms (*p* = 0.111 [2-sided] or *p* = 0.056 [1-sided]) and 40% for female worms (*p* = 0.1905 [2-sided] or *p* = 0.0952 [1-sided]), again supporting the enhanced effect of the vaccine on female worms given their increased nutritional requirements for egg production.

**Figure 5:**
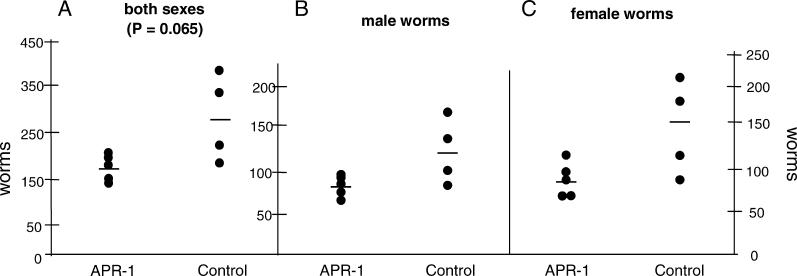
Vaccination with APR-1 Reduces Adult Worm Burdens of Dogs after Challenge Infection with Hookworms Statistically significant reduction at the *p* < 0.1 level (*p* = 0.065) in median adult worm (both sexes) burdens of dogs vaccinated with APR-1 compared to dogs that received adjuvant alone (A). Reductions are also shown when only male (B) (*p* = 0.111) and only female (C) (*p* = 0.1905) worms were considered; however, statistically significant reductions were not achieved for single sex analyses. Bars represent the median value for each group.

### Vaccination with APR-1 Protects against Anemia

Hb levels in four of the five dogs that were vaccinated with APR-1 were significantly elevated when compared with control dogs (adjuvant alone) after challenge infection (Figure 6). The median Hb concentration of vaccinated dogs for the last two time points (0 and 7 d prior to necropsy) was 12.45 g·dl^−1^ compared with 9.5 g·dl^−1^ for the control dogs that were immunized with adjuvant alone (*p* = 0.049; Mann-Whitney *U* test). A decline in Hb levels was seen in all of the control dogs after challenge infection; the decline was marked in three of the four dogs. Four of the five dogs that were vaccinated with APR-1 did not show a similar decline, and had Hb levels within (or very close to) the normal clinical range of 12–14 g·dl^−1^. One dog (C5) from the vaccinated group did become anemic (Hb concentration was 9.6 g·dl^−1^), and this animal had more female worms (120 compared with a mean of 88 female worms for the group) and more male worms (87 compared with a mean of 80 male worms for the group). However, using both Spearman and Pearson tests, we did not detect a significant correlation between worm burdens (for either or both sexes) and Hb status of the vaccinated dogs.

**Figure 6:**
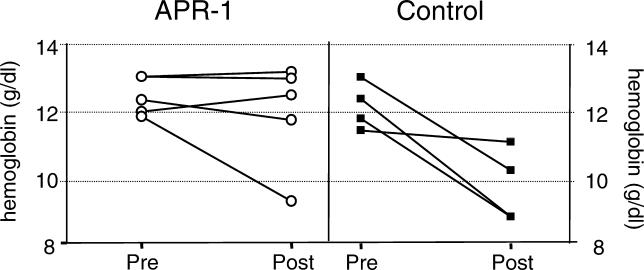
Vaccination of Dogs with APR-1 Reduces Blood Loss and Protects against Anemia Hb concentrations of vaccinated dogs were significantly (*p* = 0.049) greater than those of control dogs when blood was drawn after larval challenge (0 and 7 d before necropsy [post]) but not when blood was drawn 5 d before larval challenge (pre).

### Anti–APR-1 Antibodies Are Ingested by and Bind to the Intestine of Feeding Hookworms

The site of anatomical expression of *Ac*-APR-1 within adult hookworms has been previously reported by us to be the microvillar surface of the gut [21,23]. To determine whether vaccination of dogs induced circulating antibodies that bound to the intestinal lumen during infection, parasites were removed from vaccinated dogs, fixed, sectioned, and probed with anti-dog IgG conjugated to Cy3. Worms recovered from dogs immunized with *Ac-*APR-1 but not from dogs immunized with adjuvant alone reacted with Cy3-conjugated anti-dog IgG (Figure 7), indicating that anti–APR-1 antibodies were ingested with the blood-meal of the worm and subsequently bound specifically to the intestine of the parasite in situ.

**Figure 7:**
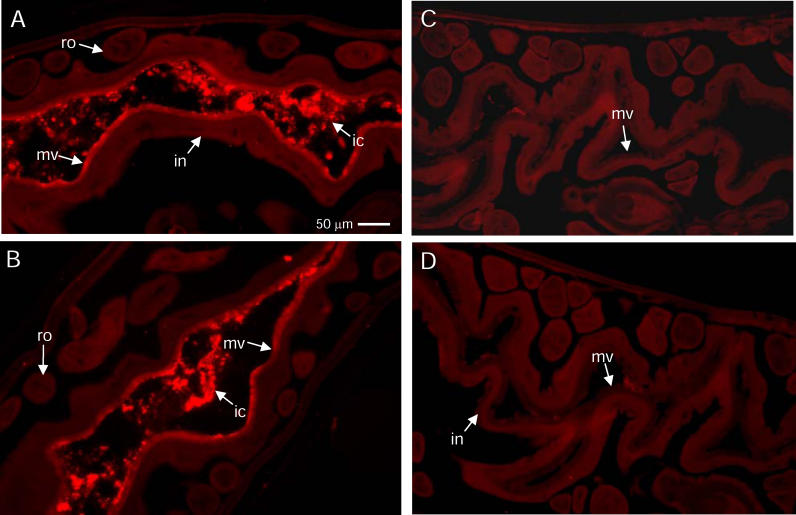
Antibodies Bind In Situ to the Intestines of Hookworms that Feed on Vaccinated Dogs Detection of antibodies that bound to the gut of worms recovered from vaccinated dogs (A and B) but not control dogs (C and D) by immunofluorescence. Binding was detected using Cy3-conjugated rabbit anti-dog IgG, allowing only detection of antibodies that had bound in situ while parasites were feeding on blood from vaccinated or control dogs. ic. intestinal contents; in, hookworm intestine; mv, intestinal microvillar surface; ro, reproductive organs.

### IgG from Dogs Vaccinated with *Ac*-APR-1 Neutralizes Proteolytic Activity In Vitro

Purified IgG from dogs that were immunized with *Ac-*APR-1 reduced the catalytic activity of the enzyme by 71%, compared with just 6% reduction when an equivalent amount of IgG from dogs immunized with adjuvant alone was assessed (Table 1). The aspartic protease inhibitor, pepstatin A, inhibits catalytic activity of APR-1 [23] and was therefore used as a positive control to obtain 100% inhibition for comparative purposes.

**Table 1:**
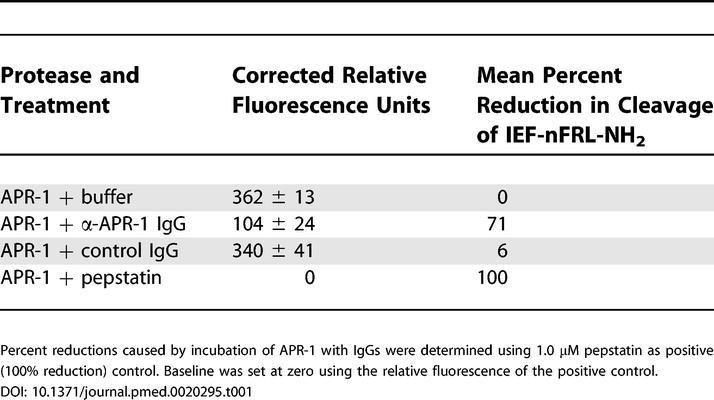
Reduction in Cleavage of the Fluorogenic Substrate o-Aminobenzoyl-IEF-nFRL-NH_2_ When 1.0 μg of Recombinant *Ac-*APR-1 Was Pre-Incubated with 0.2 μg of IgG Purified from Sera of Dogs Vaccinated with APR-1/AS03 or AS03 Alone (Control)

## Discussion

Here we describe protective vaccination of dogs with a recombinant aspartic hemoglobinase, a pivotal enzyme in the initiation of Hb digestion in the gut of canine hookworms [12,21]. We show that APR-1 provides the best efficacy thus far reported for a recombinant vaccine aimed at reducing hookworm egg counts, intestinal worm burdens, and hookworm-induced blood loss.

The vaccine efficacy of recombinant *Ac-*APR-1 expressed in baculovirus-infected insect cells was described earlier by us [24]; however, this initial vaccine trial was hampered by limited availability of the recombinant protein: Suboptimal doses were used and antibody responses (titers <10,000) were first observed just 1 wk following the third (and final) immunization, and only in some dogs. Despite the weak antibody responses, a statistically significant reduction in mean (18%, *p* < 0.05) and median (23%) hookworm burdens were observed. In addition there was a shift of adult hookworms from the small intestine to the colon [24]. However, no reduction in the mean fecal egg counts were observed, and hematologic parameters were not assessed. The improved immunogenicity of APR-1 observed in this study might also be attributed to use of the adjuvant AS03 compared with alhydrogel in the previous study. We have shown in a head-to-head comparison of a hookworm cysteine hemoglobinase formulated with different adjuvants (including alhydrogel) that AS03-formulated protein generated higher antibody titers and afforded greater protection to vaccinated dogs [22]. In this study, we show that yeast-derived APR-1 provides the best efficacy thus far reported for a recombinant vaccine aimed at reducing hookworm load and potential transmission. Moreover, we show that vaccination protects against the pathology associated with worm-induced blood loss, or hookworm disease.

Hookworms bury their anterior ends into the intestinal mucosa to feed, secreting anticoagulants to promote blood flow and stop clot formation at the site of attachment (reviewed in [28]). Numerous anticoagulant peptides have been reported from hookworms [29–31], and their combined activities result in “leakage” of blood around the attachment site and into the host intestine [32]. It is not known whether the majority of blood loss during a hookworm infection is due to leakage around the feeding site or from ingested blood that enters the parasite's alimentary canal for nutritional purposes. To address this, attempts have been made to measure blood lost from the anus of *A. caninum* (i.e., blood that has passed through the parasite's alimentary canal); varying calculations have been proposed ranging from 0.14–0.8 ml blood expelled over 24 h per adult worm (reviewed in [32]). Whatever the true figure is, significant blood loss occurs via this route, supporting the hypothesis that vaccination with APR-1 damages that parasite's intestine and results in decreased blood intake (and blood loss) by feeding worms.

The immunological parameters required for vaccine-induced protection against hookworm infection were, until recently, poorly defined. Protection against *A. caninum* by vaccination of dogs with radiation-attenuated L3 was reported many years ago [5]; however, it was not until recently that murine [8,33] and canine [34] studies revealed the protective mechanisms of the irradiated larval vaccine at a cellular level. These studies suggested that a T-helper type-2 response is induced by vaccination with irradiated L3; however the authors did not prove that a T-helper type-1 response abrogates protection. In our study reported here, dogs vaccinated with APR-1 generated strong memory responses to the recombinant antigen and did not secrete Th-2 cytokines but instead secreted IFN-γ in response to stimulation with recombinant APR-1. Moreover, the dominant antibody isotype induced by vaccination was IgG2, suggesting that a Th-1-like response was generated. Unlike the clear association between IgG2 and type I cytokines such as IFN-γ in mice and humans, little is known about this association in dogs. Experimental evidence using the canine model suggests that immune responses (Th1 versus Th2) are, however, linked to isotype production. For example, animals infected with and protected against visceral leishmaniasis (Th1 response) or *Salmonella* (also a Th1 response) mount a higher IgG2 than IgG1 response [35,36]. Our data [34] show that dogs immunized with irradiated hookworm larvae demonstrated a stronger production of IgG1 (also supported by [37]) which accompanied IL-4 production, implying a Th2 cytokine response in dogs is accompanied by the same immunoglobulin isotypes seen in humans and mice. Based on the current data, we cannot conclude that a Th-1 response to APR-1 is required to obtain protection; however, it does not inhibit the development of a protective memory response. It should also be considered that successful immunity to the different developmental stages of hookworms might require very different immune response phenotypes, not unlike those seen in schistosomiasis [38]. Further studies will explore the effects of vaccination with APR-1 formulated with different adjuvants and co-factors (e.g., cytokines) that will promote a Th2 response.

Hematophagous helminths require blood as a source of nutrients to mature and reproduce. Female schistosomes ingest 13 times as many erythrocytes and ingest them about nine times faster than male worms [39]. Moreover, mRNAs encoding Hb-degrading proteases of schistosomes are overexpressed in female worms [40]. Although similar studies have yet to be performed for hookworms, female hookworms are bigger than males and lay up to 10,000 eggs per day, implying that they have a greater metabolism and therefore greater demand for erythrocytes**.** *Ac-*APR-1 degrades Hb in the gut lumen of the worm, and it is therefore not surprising that interruption of the function of APR-1 via the action of neutralizing antibodies has a deleterious effect on the establishment of worms, particularly females and their subsequent egg production. We observed a similar (although not as pronounced) phenomenon when dogs were vaccinated with the cysteine hemoglobinase, *Ac-*CP-2, followed by challenge infection with *A. caninum* L3 [22]. Vaccination with CP-2, however, did not result in reduced adult worm burdens or reduced blood loss, essential attributes of an efficacious hookworm vaccine.

Vaccination of livestock and laboratory animals with aspartic proteases of other nematodes, as well as trematode helminths, has resulted in antifecundity/antiembryonation effects. Immunization of sheep with the intestinal brush border complex, H-gal-GP, confers high levels of protection (both antiparasite and antifecundity) against *H. contortus* and at least three different protease activities, including aspartic proteases, have been detected in this extract [16,41]. Immunization of sheep with aspartic protease-enriched fractions of *H. contortus* membranes resulted in 36% reduction in adult worms and 48% reduction in fecal egg output [17]. Vaccination of sheep with denatured *H. contortus* proteases or recombinant proteases expressed in bacteria, however, did not confer protection, suggesting that conformational epitopes are important in protection [17]. Vaccination of mice with recombinant aspartic protease of the human blood fluke, *Schistosoma mansoni,* resulted in 21%–38% reduction in adult parasites after challenge with infective cercariae; however a reduction in eggs deposited in the liver (the cause of most pathology in schistosomiasis) was not detected [42]. Protective efficacy of aspartic proteases has been observed against fungal pathogens as well. Vaccination of mice with secreted aspartic proteases of *Candida albicans,* known virulence factors in candidiasis, protected animals against a lethal challenge infection and inhibited colonization of fungi in the kidneys [43]. Moreover, passive transfer of serum from vaccinated animals conferred protection, pointing towards an antibody-mediated protective mechanism.

Almost all of the pathology and morbidity of human hookworm infection results from intestinal blood loss caused by large numbers of adult hookworms. Depending on host iron and protein stores, a range of hookworm intensities, equivalent to burdens of 40 to 160 worms, is associated with Hb levels below 11 g·dl^−1^, the World Health Organization threshold for anemia. In Tanzania, Nepal, and Vietnam where host iron stores are generally depleted, there is a direct correlation between the number of adult hookworms in the intestine and host blood loss [1,44]. Therefore the optimal hookworm vaccine will be one that either prevents L3 from developing into adult blood-feeding hookworms, or one that blocks the establishment, survival, and fecundity of the adult parasites in the intestine [3,45]. Achieving both goals will likely require a vaccine cocktail comprised of an L3 antigen, such as ASP-2 now under clinical development [46,47], and an adult gut protease, such as APR-1.

An effective hookworm vaccine need not attain 100% efficacy. Unlike many unicellular organisms that reproduce asexually within the host, nematodes need to sexually reproduce. Therefore, small numbers of adult worms will generate fewer eggs to contaminate the environment, and subsequently reduce transmission. More importantly, because hookworms are blood feeders, a partial reduction in adult worm burden equates to a decrease in pathology, notably iron-deficiency anemia [44]. Mathematical modeling of schistosomiasis in China showed that elimination of the parasite could be attained using an antifecundity vaccine that targets egg output with 75% efficacy [48], and it is likely that a similar scenario applies to long-term elimination of soil-transmitted helminths such as hookworms. An orthologue of *Ac-*APR-1 has been reported from the major human hookworm, *N. americanus* [23]. *Na*-APR-1 is structurally and antigenically very similar to *Ac-*APR-1 and also functions as a hemoglobinase [23]. For this reason, we believe that APR-1 is now the major vaccine antigen from the adult stage of the parasite, and as such, *Na-*APR-1 should undergo process development and enter into Phase I clinical trials as a vaccine for human hookworm infection. This vaccine strategy is now being implemented for a larval hookworm antigen, with Phase 1 human trials using ASP-2 formulated with Alhydrogel already underway [49]. Based on the data reported here, APR-1 may also be selected for downstream process development, manufactured under good clinical manufacturing processes, and tested in the clinic.

## Supporting Information

### Accession Numbers

The GenBank (http://www.ncbi.nlm.nih.gov/Genbank) accession numbers for the gene products mentioned in this paper are *Ac-*APR-1 (U34888) and *Na*-APR-1 (AJ245459).

**Patient Summary**

**Background**

Hookworms are parasites of the intestines. They can infect many animals, including dogs, cats, and people. Worldwide, about one person in five has a hookworm infection. Most of these one billion people live in tropical countries. Hookworm is not spread from person to person, because at one stage of its lifecycle, the parasite needs to be in the soil. In areas where hookworm is common, people who have contact with soil that contains human feces are at high risk of infection; because children play on soil and often go barefoot, they have the greatest risk. Infection leads to blood loss and a decrease in the amount of iron, and this causes anemia (i.e., because of a lack of iron, the blood cannot carry oxygen efficiently). There are effective drugs to treat the infection, but they do not prevent the patient from becoming re-infected. Making a vaccine against hookworm is therefore a priority. Some vaccines for use in animals have already been developed, but their effectiveness is limited to one stage of the hookworm's lifecycle. The aim is to find a vaccine that works against more than one of the stages that the parasite passes through in its lifecycle.

**What Did the Researchers Do and Find?**

The researchers focused on two enzymes the parasite needs in order to live. Building on earlier research and using a species of hookworm that affects dogs, the researchers aimed to make these enzymes the “target” of a vaccine. They first vaccinated dogs, then infected them with hookworm. These dogs had fewer parasites than dogs that had not been vaccinated. Most importantly, vaccinated dogs were protected against blood loss, and most did not develop anemia. Laboratory tests confirmed that the target enzymes had been damaged.

**What Do These Findings Mean?**

This is the best result so far for a hookworm vaccine used in dogs. The authors believe that, as well as reducing parasite numbers, the vaccine reduces the ability of the parasite to take in blood, which would explain the reduction in anemia. The researchers have called for trials to begin with a vaccine targeted against similar enzymes in the species of hookworm that most commonly affects humans.

**Where Can I Get More Information Online?**

The US Centers for Disease Control have a fact sheet on hookworm:

http://www.cdc.gov/ncidod/dpd/parasites/hookworm/factsht_hookworm.htm.

The Sabin Vaccine Institute has an overview of the Human Hookworm Vaccine Initiative:

http://www.sabin.org/hookworm.htm.

## Abbreviations

APR-1: *Ancylostoma caninum* aspartic protease 1
AS03: GlaxoSmithKline Adjuvant System 01
ELISA: enzyme-linked immunosorbent assay
epg: eggs per gram of feces
Hb: hemoglobin
L3: third stage larvae
